# AGE DIFFERENCES IN THE TEMPORAL PATTERNS OF SELF-CONTINUITY AND TEMPORAL DISCOUNTING

**DOI:** 10.1093/geroni/igae098.3419

**Published:** 2024-12-31

**Authors:** Yi Lu, Corinna Loeckenhoff

**Affiliations:** Cornell University, ITHACA, New York, United States; Cornell University, Cornell University, New York, United States

## Abstract

Self-continuity, the perceived connectedness with past and future selves, varies across the life span. Previous research found that both past and future self-continuity are positively associated with age and that such effects are more pronounced for more distant time intervals (Rutt & Löckenhoff, 2016). Questions remain about the appropriate mathematical modeling of such patterns and potential associations between age differences in self-continuity and temporal discounting, the tendency to devalue future outcomes. In response, the present study assessed both past and future self-continuity as well as temporal discounting across four temporal distances (0.5, 1, 5, 10 years) in a U.S. adult life-span sample (N=461, aged 18–96, M = 51.38, SD =19.54, 52% female, 65% Non-Hispanic White). When compared to linear and exponential models, hyperbolic models provided the best fit for both self-continuity and temporal discounting. Subsequent analyses therefore focused on hyperbolic slope parameters (k) derived for each participant. Chronological age was negatively associated with k for both self-continuity and temporal discounting, indicating that, with age, the perceived connectedness with past/future selves and the perceived value of delayed rewards show fewer decrements with increasing temporal distance. However, although k parameters for past/future self-continuity were significantly associated with each other, they were not significantly associated with temporal discounting. Moreover, age differences in temporal discounting remained significant after controlling for age differences in past/future self-continuity. The present study extends our understanding of previously reported age differences in self-continuity and temporal discounting but suggests that they cannot be traced to the same underlying mechanism.

